# Supplementary material for: A 16S RNA Analysis of Yangzhou Geese with Varying Body Weights: Gut Microbial Difference and Its Correlation with Body Weight Parameters
Source: Animals (Basel). 2024 Jul 12;14(14):2042. doi: 10.3390/ani14142042 (PMC11273403; doi:10.3390/ani14142042)
Supplement: Supplementary file 1 [file animals-14-02042-s001.zip › animals-3091417 supplementary.pdf]

Table S1. Data quality control.

Group	Sample	RawPE	Combined	Qualified	No chime	Base (nt)	Avglen (nt)	GC	Q20	Q30
L Group	L.341	107699	107024	104777	87790	36445206	415.14	52.08%	98.50%	95.02%
	L.145	103154	102680	100678	84231	35151171	417.32	51.51%	98.44%	94.83%
	L.390	110175	109657	107649	95432	39368118	412.53	52.67%	98.57%	95.24%
	L.267	104272	103737	101522	88985	37256405	418.68	51.15%	98.50%	95.00%
	L.81	106413	105790	103804	83894	35132986	418.78	51.04%	98.48%	94.98%
	L.3	106204	105649	103594	92982	38779512	417.06	51.87%	98.46%	94.89%
S Group	S.652	106398	105809	103701	86668	36348540	419.4	51.00%	98.54%	95.11%
	S.189	112222	111430	109133	92639	38771328	418.52	51.26%	98.39%	94.78%
	S. z029	104232	103633	101663	85626	35675130	416.64	51.70%	98.49%	95.04%
	S.2	113516	112955	110560	100685	42098800	418.12	51.03%	98.49%	95.03%
	S.30	104337	103693	101626	90668	37787890	416.77	52.26%	98.44%	94.88%
	S.841	110038	109488	107378	98372	41145329	418.26	51.69%	98.52%	95.08%

Table S2. Alpha Diversity Index Statistics.

Sample_Name	chao1	dominance	goods_coverage	observed_features	pielou_e	shannon	simpson
L.81	628.000	0.013	1.000	628	0.799	7.427	0.987
L.145	685.871	0.012	1.000	682	0.796	7.494	0.988
L.3	606.045	0.016	1.000	604	0.783	7.237	0.984
L.341	624.000	0.018	1.000	619	0.789	7.319	0.982
L.390	539.125	0.013	1.000	538	0.803	7.286	0.987
L.267	614.875	0.014	1.000	613	0.803	7.436	0.986
S.89	800.000	0.013	1.000	795	0.787	7.582	0.987
S.z029	812.222	0.013	1.000	802	0.799	7.707	0.987
S.30	653.059	0.017	1.000	643	0.760	7.087	0.983
S.2	807.404	0.020	1.000	806	0.773	7.467	0.980
S.652	654.000	0.032	1.000	651	0.714	6.676	0.968
S.841	589.200	0.031	1.000	588	0.721	6.633	0.969

Table S3. Averaged relative abundance of the top 10 main intestinal microbial colonies at the phylum level.

Taxonomy	L Group	S Group
Firmicutes	0.446222	0.395368
Bacteroidota	0.467808	0.528333
Desulfobacterota	0.045901	0.042135
Actinobacteriota	0.02688	0.014766
Spirochaetota	0.001702	0.011776
Deferribacterota	0.006695	0.00062
Fusobacteriota	0.000792	0.002189
Proteobacteria	0.001311	0.002259
Campylobacterota	0.00079	0.000292
Euryarchaeota	0	0.000901

Note: Average relative abundance.

Table S4. Averaged relative abundance of the top 10 main intestinal microbial colonies at the genus level.

Taxonomy	L Group	S Group
Bacteroides	0.224951	0.310604
PrevotellaceaeGa6A1_group	0.084755	0.02836
Subdoligranulum	0.05307	0.037864
Desulfovibrio	0.045195	0.041778
[Ruminococcus]torques_group	0.02076	0.040544
Romboutsia	0.006476	0.019338
Treponema	0.001265	0.011057
Anaerofilum	0.005652	0.014531
Peptococcus	0.008682	0.020327

Note: Average relative abundance.
